# Effects of increasing intranuclear calcium levels via MCU inhibition on mouse and human PSC-derived cardiomyocyte differentiation and maturation

**DOI:** 10.1093/stcltm/szag021

**Published:** 2026-04-27

**Authors:** Hyun Ju Seo, Ju-Young Kim, Hyun-Jai Cho, Joo-Eun Lee, Sang-Beom Bang, Mika Jeon, Hyang-Ae Lee, Yoo-Jeong Shin, Han‑Mo Yang, Hyo-Soo Kim

**Affiliations:** Department of Molecular Medicine and Biopharmaceutical Sciences, Graduate School of Convergence Science and Technology, Seoul National University, Seoul 03080, Korea; New Drug Development Division, K-BioCELF Inc., Suwon 16229, Korea; Interdisciplinary Program in Stem Cell Biology, College of Medicine, Seoul National University, Seoul 03080, Korea; Department of Molecular Medicine and Biopharmaceutical Sciences, Graduate School of Convergence Science and Technology, Seoul National University, Seoul 03080, Korea; Interdisciplinary Program in Stem Cell Biology, College of Medicine, Seoul National University, Seoul 03080, Korea; Department of Internal Medicine, Seoul National University Hospital, Seoul 03080, Korea; Interdisciplinary Program in Stem Cell Biology, College of Medicine, Seoul National University, Seoul 03080, Korea; Department of Molecular Medicine and Biopharmaceutical Sciences, Graduate School of Convergence Science and Technology, Seoul National University, Seoul 03080, Korea; Department of Molecular Medicine and Biopharmaceutical Sciences, Graduate School of Convergence Science and Technology, Seoul National University, Seoul 03080, Korea; Center for Biomimetic Research, Korea Institute of Toxicology (KIT), 34114 Daejeon, Korea; School of Korea Institute of Toxicology, University of Science and Technology (UST), 34113 Daejeon, Korea; Faculty of Medicine and Health, University of Sydney, Sydney, NSW 2006, Australia; Department of Internal Medicine, Seoul National University Hospital, Seoul 03080, Korea; Department of Molecular Medicine and Biopharmaceutical Sciences, Graduate School of Convergence Science and Technology, Seoul National University, Seoul 03080, Korea; Interdisciplinary Program in Stem Cell Biology, College of Medicine, Seoul National University, Seoul 03080, Korea; Department of Internal Medicine, Seoul National University Hospital, Seoul 03080, Korea

**Keywords:** 7-aminoindole, mitochondrial calcium uniporter (MCU), CaMK–CREB signaling, maturation, human induced pluripotent stem cell-derived cardiomyocyte

## Abstract

Background

Cardiovascular diseases remain the leading cause of death worldwide, accounting for approximately 19.41 million deaths annually. Human-induced pluripotent stem cell-derived cardiomyocytes (hiPSC-CMs) hold great promise for disease modeling and therapy. However, variability in differentiation efficiency and maturation state remains a significant challenge. Calcium signaling plays a pivotal role in cardiac differentiation and maturation, with mitochondrial calcium dynamics closely linked to transcriptional activation. Recent evidence suggests that closure of the mitochondrial permeability transition pore promotes cardiomyocyte differentiation by stabilizing calcium homeostasis and reducing oxidative stress.

Methods

We investigated the effect of 7-aminoindole (7-AI), a mitochondrial calcium uniporter inhibitor, on cardiomyocyte differentiation. Mouse embryonic stem cells and hiPSCs were treated with 7-AI during the cardiac progenitor stage (day 4). Nuclear calcium levels, Ca2+/calmodulin-dependent protein kinase (CaMK) activation, CREB phosphorylation, and cardiac marker expression were assessed.

Results

7-AI increased nuclear calcium levels, activated CaMK and CREB, and upregulated cardiac-specific markers, including cTnT, α-SA, and MYH6/7. The resulting cardiomyocytes exhibited improved sarcomere organization and enhanced contractility. Moreover, CREB-deficient cells failed to exhibit these effects, confirming that CREB activation is essential for 7-AI-mediated cardiac maturation.

Conclusions

Collectively, our findings demonstrate that inhibition of mitochondrial calcium influx redistributes calcium to the nucleus, thereby activating the CaMK–CREB signaling axis and promoting cardiomyocyte differentiation. Targeting mitochondrial calcium handling at the cardiac progenitor stage provides a mechanistic and pharmacological approach to enhance the structural and functional maturation of cardiomyocytes for therapeutic application.

## Graphical abstract

**Graphical Abstract:**
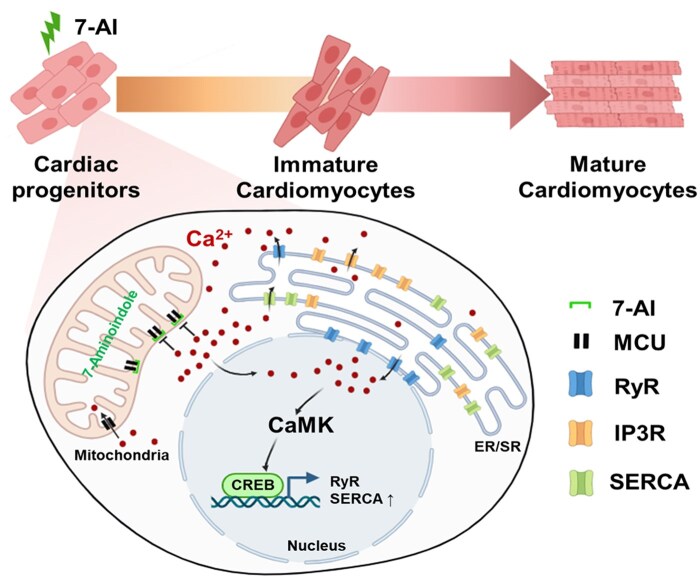

Significance statement Efficient and reproducible generation of mature cardiomyocytes from pluripotent stem cells remains a major challenge in cardiac regenerative medicine. This study demonstrates that transient inhibition of mitochondrial calcium uptake during the cardiac progenitor stage enhances cardiomyocyte differentiation and structural maturation by redistributing calcium to the nucleus and activating the CaMK–CREB signaling axis. These findings identify mitochondrial calcium handling as a key upstream regulator of transcriptional remodeling during cardiac lineage specification and provide a mechanistic and pharmacological strategy to improve the functional maturation of stem cell-derived cardiomyocytes for disease modeling and therapeutic applications.

## Introduction

Cardiovascular diseases are the leading cause of mortality worldwide, necessitating the exploration of underlying mechanisms and clinical manifestations and the development of innovative therapeutic strategies for these diseases. Human-induced pluripotent stem cell-derived cardiomyocytes (hiPSC-CMs) are valuable tools providing a robust platform for cardiac research, disease modeling, and drug screening.^1–3^ However, improving the efficiency of hiPSC-CM differentiation is a major challenge. Therefore, in this study, we aimed to enhance the efficiency of hiPSC-CM differentiation using a novel compound to modulate the intracellular calcium dynamics.

Our study was designed to investigate the mechanistic contribution of mitochondrial calcium flux to cardiac lineage specification rather than to optimize differentiation yield. To ensure that potential regulatory effects of 7-AI could be clearly discerned, we intentionally employed unoptimized conditions with modest baseline efficiency as a sensitized model system. This approach is commonly used in mechanistic differentiation studies, where high baseline efficiency may mask subtle regulatory effects.^4–6^

Closure of the mitochondrial permeability transition pore (mPTP), which promotes mitochondrial maturation and CM differentiation, reduces the reactive oxygen species (ROS) levels and modulates the calcium dynamics in early embryonic CMs.^7^ As mPTP closure influences calcium homeostasis, we hypothesized that the initial inhibition of calcium influx is the primary mechanism protecting mitochondria and driving stem cell differentiation, whereas the observed reduction in ROS levels is a downstream effect of altered calcium signaling, as suggested in a previous report.^8^ Therefore, calcium signaling, rather than ROS, is the key regulator of CM maturation.^9^^,^^10^

Ca^2+^/calmodulin-dependent protein kinase (CaMK) integrates intracellular calcium signals to regulate various cellular processes, such as cell differentiation.^11–13^ During hiPSC-CM maturation, CaMK coordinates calcium signaling among the cytosol, endoplasmic/sarcoplasmic reticulum, and mitochondria, influencing various CM-specific functions.^14–16^ We previously demonstrated that 7-aminoindole (7-AI), a compound inhibiting mPTP opening and mitochondrial calcium influx, provides cardioprotection by preserving the mitochondrial membrane potential and suppressing ROS production.^13^ Based on these findings, we hypothesized that 7-AI enhances hiPSC-CM differentiation by modulating the mitochondrial calcium uptake and that treatment with 7-AI during the cardiac progenitor cell stage increases the cytosolic and nuclear calcium levels by inhibiting the mitochondrial calcium uniporter (MCU), as suggested in previous reports.^17^^,^^18^ Calcium redistribution possibly activates CaMK and the transcription factor, cAMP response element-binding protein (CREB), which together promote the expression of cardiac-specific genes, including those encoding ryanodine receptors, inositol 1,4,5-trisphosphate receptors, and sarco/endoplasmic reticulum calcium ATPase (SERCA).^19^^,^^20^ Consistent with the proposed mechanism, our comparative analyses of wild-type and CREB-deficient cells revealed that functional CREB was essential for CM maturation by increasing the CM marker (cardiac troponin T [cTnT], α-actinin, and MYH6/7) levels and supporting proper myofibrillar organization.

This study showed that targeting calcium dynamics via MCU inhibition during the cardiac progenitor cell stage (day 4) significantly improved the CM differentiation efficiency, providing insights into the fundamental mechanisms governing cardiac differentiation and presenting a novel strategy to enhance cardiac differentiation. Comprehensive understanding of the association between calcium distribution and cardiac differentiation will facilitate the efficient generation of functional CMs for various therapeutic applications.

## Methods

### Material: necrosis inhibitor

A novel necrosis inhibitor—a 7-amino-indole chemical—was developed by LG Chem at the LG Chem Life Science R&D Campus in Daejeon, Korea (http://www.rnd.lgchem.com/global/main). The study material, referred to as NecroX, is available upon reasonable request. Based on preliminary experiments with various compounds in the NecroX series, we selected NecroX-7 ((tetrahydropyran-4-yl)-[2-phenyl-5-(1,1-dioxothiomorpholin-4-yl) methyl-1H-indol-7-yl]amine; C_25_H_32_N_4_O_4_S_2_) for further evaluation.

### hiPSC culture and differentiation

hiPSCs reprogrammed from newborn foreskin fibroblasts (GSC-3006G; AMS Biotechnology [GlobalStem], Abingdon, UK) using the 4 Yamanaka factors were cultured using STO (SIM; ATCC number: CRL-1503) on the Dulbecco’s modified Eagle’s medium/nutrient mixture F12 Glutamax (10565-018; Thermo Fisher Scientific) supplemented with knockout serum replacement, 10 mM non-essential amino acids, 200 mM l-glutamine, 55 mM β-mercaptoethanol, and 10 ng/mL human recombinant bFGF.

CM differentiation of iPSCs was performed as described by Lian et al., with some modifications. Directed CM differentiation from human pluripotent stem cells by modulating Wnt/b-catenin signaling under fully defined conditions; Nature protocols, 2013, 162, Vol. 8 No. 1. hiPSC colonies were detached using dispase (17105-041; Thermo Fisher Scientific) and dissociated into single cells. Then, 1.5 × 10^6^ hiPSCs were seeded on Matrigel (354277)-coated 35-mm dishes, grown on mTeSR1 (#85851; STEMCELL Technologies) until reaching 100% confluency, and subjected to cardiac differentiation. The following chemicals were sequentially administered: 6 μm CHIR99021 (252917-06-9; Cayman) for the first 2 days, followed by 10 μm recombinant human/mouse/rat activin A (338-AC; R&D Systems) and 20 μm recombinant human bFGF (13256029; Thermo Fisher Scientific) the next day, and 5 μm IWR1 (I0161; Sigma-Aldrich) 3 days after that. The medium was replaced with the Roswell Park Memorial Institute-1640 medium (11875-085; Thermo Fisher Scientific) supplemented with B27 supplement (minus insulin) once every 2 days until the CMs contracted (Table S2). Subsequently, 7-AI (5 and 100 nM) was administered one day before cell differentiation initiation. MCU inhibitor RuR (5 μm) was also administered at the same time as 7-AI.

### Cardiomyocyte characterization

Spontaneous beating of the differentiated cardiomyocytes was recorded using a phase-contrast microscope. Beating area (%) and contraction rate (bpm) were analyzed from 3-minute recordings using ImageJ according to the experimenter’s method.

Cardiomyocytes were stained for sarcomeric proteins and imaged by confocal microscopy. Cell size (μm) and length (μm) were quantified cells using ImageJ. Statistical significance was evaluated using 1-way ANOVA, with *P* < .05 considered significant.

### FLUO-4 assay

hiPSC-CMs cultured in 35-mm dishes were stained with 1 μm FLUO-4 (F14201; Thermo Fisher Scientific, Invitrogen) and 2 mM probenecid for 15 minutes at 37 °C in a 5% CO_2_ incubator. After FLUO-4 staining, the medium was replaced with PBS containing 1% fetal bovine serum (FBS), and the cells were incubated at room temperature for 20 minutes. Subsequently, the medium was switched to Tyrode’s solution containing glucose and calcium to measure the calcium changes. To determine the calcium levels in CMs, 1 mM caffeine was applied and calcium signal changes were observed using the A1 confocal laser microscope (Nikon) via time-lapse confocal imaging. Images were captured every 2 seconds for 10 minutes. Fluorescence intensity data for each cell was acquired using the NIS-Elements C software (Nikon), and Δ*F/F* was calculated from this data. Δ*F/F* of calcium signals were graphed, and maximum intensity of calcium signals, signal rise rate, and time to reach 90% of the maximum calcium signal were quantified.

To assess the intracellular calcium signaling changes during CM differentiation, iPSCs were seeded in a 35-mm µ-Dish (ibidi) and cultured until they reached the desired confluency. After incubating with 1 μm FLUO-4 and 2 mM probenecid in 5% CO_2_ incubator at 37 °C for 15 minutes, the cells were incubated again at room temperature for 15 minutes. Then, the cells were washed with PBS, and the medium was replaced with PBS containing 1% FBS. To assess the intracellular calcium flux, 6 μm CHIR99021, compound used in the early stage of CM differentiation, was applied to stimulate calcium movement in cells.

### Nuclear and cytoplasmic protein extraction

Vehicle and hiPSC-CM groups treated with 7-AI (5 and 100 nM) from day 1 of differentiation were harvested on day 6. To extract the nuclear and cytoplasmic proteins from differentiated CMs, the cells were washed with PBS, transferred to a 15-mL tube, and centrifuged at 190 g for 5 minutes at 4 °C. The supernatant was aspirated, and the cells were resuspended in 1 mL of cold PBS in a 1.5-mL tube, followed by centrifugation at 3500 rpm for 5 minutes. The pellet was resuspended in 200 µL (per 100 mm dish) of cold buffer A and vortexed. After allowing the lysate to swell on ice for 20 minutes, it was passed through a 1-mL syringe fitted with an 18-21-gauge needle 3-4 times, and cell lysis was confirmed via trypan blue staining. The lysate was further centrifuged at 9000 rpm for 15 minutes, and the resulting supernatant containing the cytosolic proteins was carefully collected. The nuclear pellet was recovered, resuspended in 20 µL of cold buffer B, and incubated on ice for 20 minutes. The lysate was centrifuged at 15 000 rpm for 5 minutes at 4 °C, and the supernatant containing the nuclear proteins was transferred to a new tube. Western blotting was performed to analyze the proteins, and concentrations of the nuclear/cytosolic proteins were determined using the BCA protein assay kit. Buffer A contained 10 mM HEPES-KOH (pH 7.9), 1.5 mM MgCl, 10 mM KCl, 0.2 mM ethylenediaminetetraacetic acid, 0.5 mM dithiothreitol (freshly added), and 0.2 mM phenylmethylsulfonyl fluoride (freshly added). Buffer B contained 20 mM HEPES-KOH (pH 7.9), 1.5 mM MgCl_2_, 25% glycerol, 0.5 mM dithiothreitol (freshly added), and 0.2 mM phenylmethylsulfonyl fluoride (freshly added).

Western blotting analysis was performed to determine the transcription factor expression levels using the phospho-CREB (Ser133; 87G3) rabbit monoclonal (#9198; Cell Signaling Technology), CREB (48H2) rabbit monoclonal (#9197; Cell Signaling Technology), anti-SERCA2 (#2861; Abcam), anti-NFATc1 (#MA3024; Thermo Fisher), and anti-lamin A/C (#2032S; Cell Signaling technology) antibodies.

### Lentiviral transduction of hiPSC-CMs

Lentiviral transduction was performed on day 4 of hiPSC-CM differentiation. Lentivirus, prepared according to the manufacturer’s protocol, and polybrene (1 μg/mL) were used for transduction over 2-3 days. TRC *CREB1* shRNA vector was constructed by Horizon (USA).

### Transfection of the calcium indicator vector into hiPSC-CMs

Calcium indicator vectors CMV-NLS-R-GECO and CMV-mito-GEM-GECO1 were kindly gifted by R. E. Campbell (plasmids #32462 and #32461; Addgene).^13^ On day 12 of hiPSC-CM differentiation, the cells were washed with PBS, and the medium was replaced with the Opti-MEM Reduced Serum Medium (Thermo Fisher Scientific). Plasmid transfection was performed using the Lipofectamine 2000 Transfection Reagent (Thermo Fisher Scientific), according to the manufacturer’s recommended protocol. Plasmid DNA (5 µg) was diluted in 125 µL Opti-MEM and gently mixed. In a separate tube, 12.5 µL of Lipofectamine 2000 Reagent was added to 125 µL Opti-MEM, mixed gently, and incubated at room temperature for 5 minutes. Then, the plasmid DNA dilution was added to the Lipofectamine 2000 dilution, mixed gently, and incubated at room temperature for 20 minutes to allow the formation of the plasmid DNA–Lipofectamine complex. After 20 minutes, the complex was applied dropwise to the cells, gently mixed by rocking the dish, and incubated at 37 °C. After 48 hours, the cells were first incubated with 1 μm FLUO-4 and 2 mM probenecid in a 5% CO_2_ incubator at 37 °C for 15 minutes and then at room temperature for 15 minutes. After washing with PBS, the medium was replaced with PBS containing 1% FBS. The cells were treated with 2 μm thapsigargin and 100 nM 7-AI or PBS as a vehicle, and calcium signaling was examined. The cells were treated with 50 μm tert-butylhydroquinone (tBH) (Sigma-Aldrich) for strong calcium stimulation. Finally, calcium signaling and fluorescence intensity analyses were conducted as described above.

### Statistical analyses

Data are represented as the mean ± standard error of the mean. Statistical analyses were conducted via one-way analysis of variance using the GraphPad Prism 5 software. Statistical significance was set at *P* < .05.

**Ethics Statement ** 

The human induced pluripotent stem cells (hiPSCs) used in this study were generated from Nuff (newborn foreskin fibroblast) cells (cat. no. AMS.GSC-3006G, AMS Biotechnology), a commercially available cell line. According to the supplier, the Nuff cells were obtained with informed consent from donors and in compliance with ethical guidelines. The reprogramming factors OCT4, SOX2, KLF4, and cMYC were introduced using lentiviruses (Takahashi et al., 2007). No additional ethics approval was required for this study, as no new human samples were collected. All animal experiments were approved by the Institutional Animal Care and Use Committee in Seoul National University Hospital (SNUH-IACUC).

## Results

### 7-AI enhances cardiac differentiation efficiency in mouse embryonic stem cells

**Figure 1:**
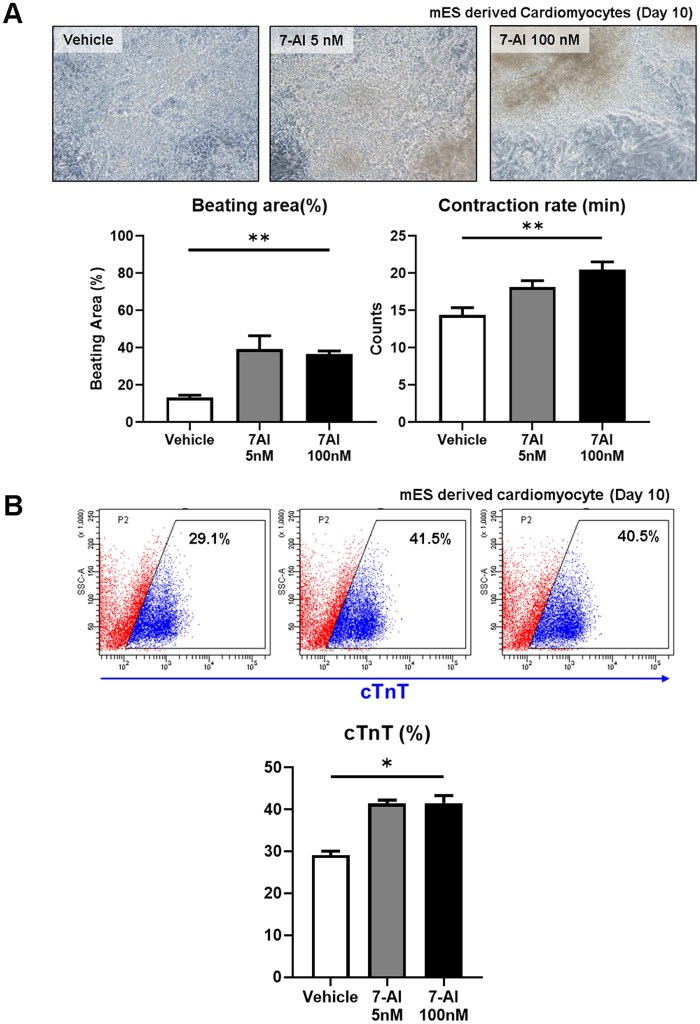
7-Aminoindole (7-AI) treatment enhances the mES cardiac-derived differentiation efficiency. (A) 7-AI treatment increased the beating area and duration of mESC-derived cardiomyocytes. (B) Flow cytometry analysis showing an increase in cardiac Troponin T-expressing cells in mESC-derived cardiomyocytes through 7-AI treatment.

We investigated the effects of 7-AI on CM differentiation using mESCs, which are relatively easy to differentiate. mESCs were treated with 7-AI (5 and 100 nM) during CM differentiation, and differentiation efficiency was compared with that of the vehicle group. Notably, 7-AI-treated groups exhibited significantly higher beating rates and more than twice the contractile area than the vehicle group (Figure 1A). Furthermore, on day 10 post-differentiation, CM-specific marker cTnT levels were significantly elevated in the 7-AI-treated groups, indicating that 7-AI increased the CM differentiation efficiency (Figure 1B).

### 7-AI promotes the maturation of mESC-derived cardiomyocytes

**Figure 2:**
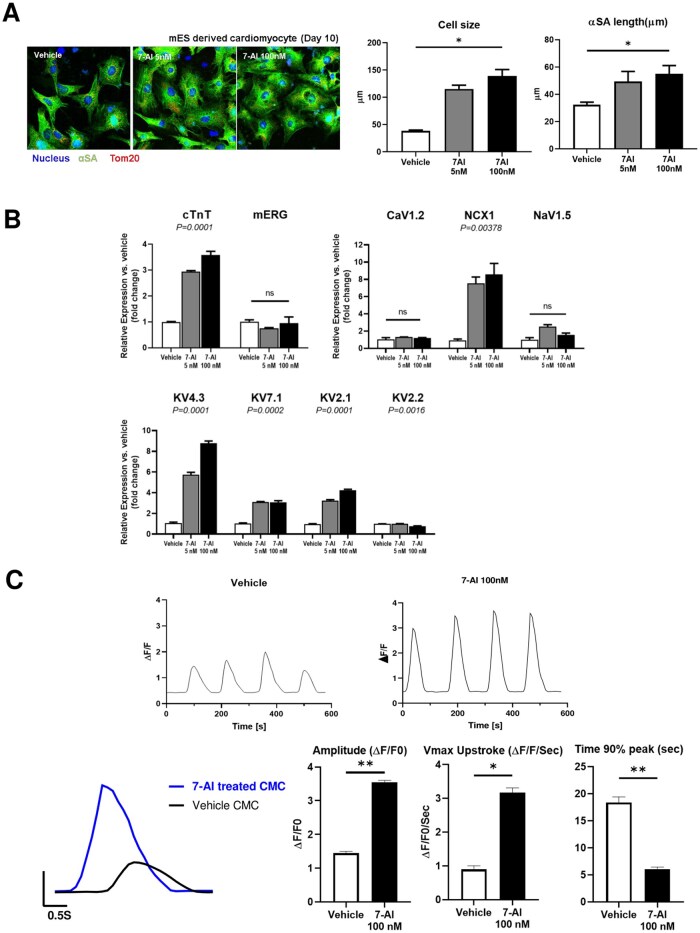
7-Aminoindole (7-AI) plays a role in increasing the maturity of cardiomyocytes during mESC-derived differentiation. (A) 7-AI treatment enhanced cardiomyocyte size by increasing the length of α-sarcomeric actinin (α-SA). (B) Real-time PCR analysis demonstrated increased expression of cardiomyocyte markers and ion channels associated with mature cardiomyocytes upon 7-AI treatment. (C) Calcium kinetics of mature cardiomyocytes, assessed using FLUO-4, revealed enhanced calcium-handling properties in 7-AI-treated cardiomyocytes.

Next, genetic and functional maturation of differentiated CMs treated with 7-AI was evaluated using α-sarcomeric actin (α-SA), a key sarcomeric structural protein. α-SA is specifically expressed in striated muscle tissues, such as CMs, making it a valuable marker to assess the functional and structural maturation of CMs. Immunofluorescence staining for α-SA, followed by length measurement revealed that the average length of α-SA was significantly longer in the 7-AI-treated groups than in the control group. Furthermore, CM size increased in a concentration-dependent manner in the 7-AI-treated groups relative to that in the vehicle group (Figure 2A).

Expression levels of ion channel genes playing key roles in the electrical and mechanical functions of mature CMs were also examined. Levels of ion channel genes *KV4.3*, *NCX1*, and *NaV1.5* were higher in the 7-AI-treated groups than in the vehicle group, although no significant differences were observed in the expression levels of *mERG*, *KV2.2*, and *CaV1.2* (Figure 2B).

Calcium kinetics analysis using Fluo-4 revealed that differentiated CMs treated with 100 nM 7-AI exhibited more than double the amplitude (Δ*F/F*) of the vehicle CMs during beating. Additionally, the treated group exhibited Vmax upstroke (Δ*F/F* per second) more than 3-fold higher than that of the vehicle group. Therefore, 7-AI-treated CMs exhibited high intracellular calcium fluctuations and rapid calcium release and uptake, showing enhanced contraction speed and efficiency. Furthermore, time to 90% peak(s) in the 7-AI-treated CM group was reduced by more than 3-fold compared to that in the control group, indicating efficient calcium release in the 7-AI-treated CM group (Figure 2C).

Collectively, these results suggest that 7-AI treatment during CM differentiation promotes the activation of ion channels regulating calcium release and uptake in calcium storage compartments, thereby supporting CM differentiation into functionally mature cells.

### 7-AI enhances cardiac differentiation efficiency in human-induced pluripotent stem cells

**Figure 3:**
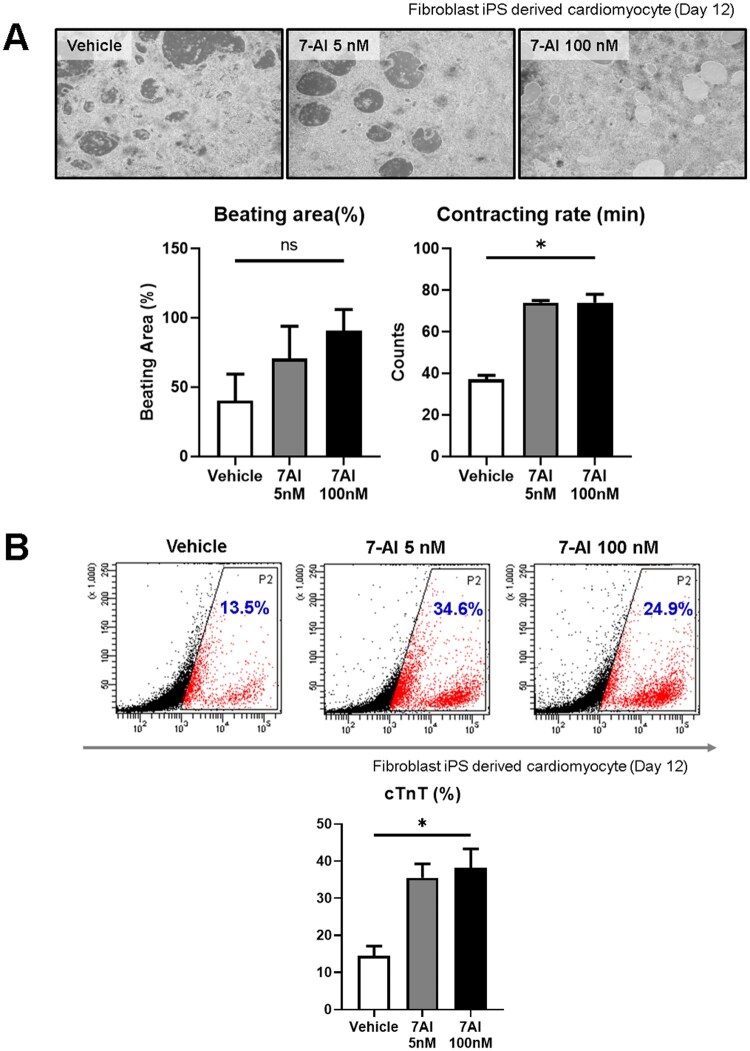
7-Aminoindole (7-AI) treatment enhances the hiPSC cardiac-derived differentiation efficiency. (A) 7-AI treatment increased the beating area and contraction time of hiPSC-derived cardiomyocytes. (B) Flow cytometry analysis showing an increase in cTnT-expressing cells in iPSC-derived cardiomyocytes following 7-AI treatment.

Next, we examined the effects of 7-AI on CM differentiation using hiPSC-CMs. During differentiation, human fibroblast-derived iPSCs were treated with 7-AI (5 and 100 nM). Notably, 7-AI-treated groups exhibited significantly stronger contractions and larger beating areas than the vehicle group (Figure 3A). To evaluate whether these effects could be attributed to other mitochondrial calcium-handling pathways, we additionally assessed the impact of cyclosporin A (CsA), a well-established inhibitor of the mPTP, on beating activity. CsA treatment increased the beating rate compared with the vehicle control, indicating that mPTP inhibition partially promotes functional maturation.^21^ However, the beating activity induced by CsA did not reach the levels achieved by optimal 7-AI concentrations, despite effective mPTP inhibition. These results suggest that while mPTP modulation contributes to CM functional enhancement, the superior effects of 7-AI are not solely mediated by mPTP inhibition but likely involve distinct mechanisms, such as MCU-dependent intracellular calcium redistribution (Figure S1B). Furthermore, cTnT levels were elevated in the 7-AI-treated groups, indicating that 7-AI increased the CM differentiation efficiency (Figure 3B).

### 7-AI promotes the maturation of hiPSC-derived cardiomyocytes

**Figure 4:**
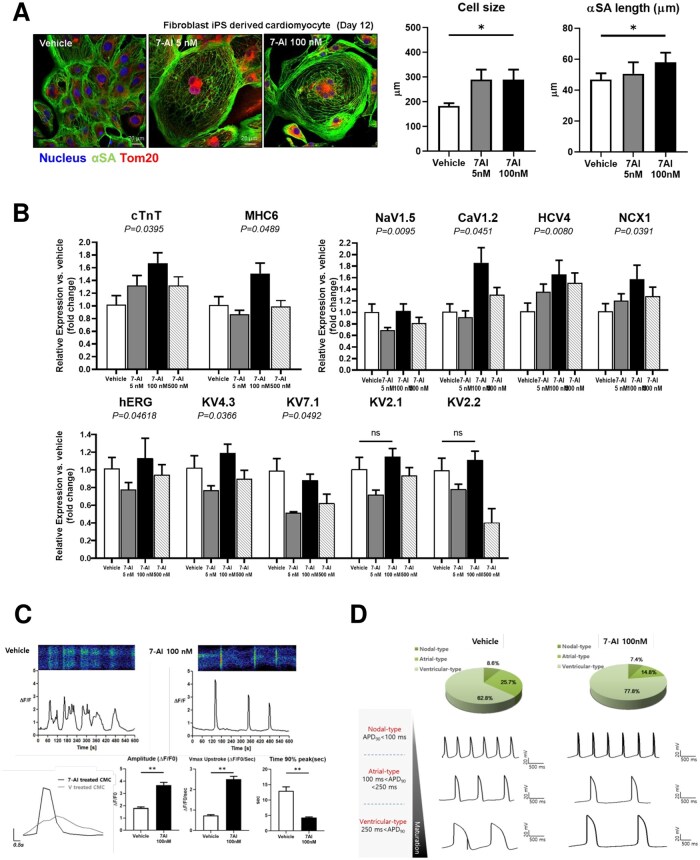
7-Aminoindole (7-AI) plays a role in increasing the maturity of cardiomyocytes during hiPSC-derived cardiomyocyte differentiation. (A) 7-AI treatment increases cardiomyocyte size by elongating α-SA. (B) Real-time PCR analysis showing the upregulation of cardiomyocyte markers and ion channels associated with mature cardiomyocytes in response to 7-AI treatment. (C) FLUO-4-based calcium imaging indicates enhanced calcium kinetics in 7-AI-treated cardiomyocytes. (D) Effects of 7-AI on action potential subtypes in hiPSC-CMs.

Structural maturity of hiPSC-CMs was examined via α-SA staining. Total cell size was significantly larger and sarcomere length was significantly longer in the 7-AI-treated group than in the vehicle group (Figure 4A). To evaluate genetic maturation, expression levels of the CM-specific markers, such as *cTnT* and *MHC6*, and genes encoding ion channels characteristic of mature CMs were analyzed. Upon treatment with 7-AI (5, 100, and 500 nM), highest increase in *cTnT* and *MHC6* levels was observed in the 100 nM 7-AI-treated group. Analysis of ion channel gene expression revealed that the 7-AI-treated hiPSC-CMs exhibited significantly higher levels of *Kv4.3* and *NCX1* (similar to mESC-CMs) as well as *hERG*, *Kv2.2*, and *CaV1.2* (different from mESC-CMs) than the vehicle CMs (Figure 4B).

In the calcium kinetics analysis of hiPSC-CMs, 100 nM 7-AI-treated group exhibited a higher amplitude (Δ*F/F*) and Vmax upstroke (Δ*F/F* per second) than the vehicle group, indicating enhanced contraction speed and calcium handling efficiency. Furthermore, time to 90% peak(s) was reduced in the 7-AI-treated group, indicating rapid calcium release. Overall, 7-AI-treated groups exhibited more stable and efficient excitation–contraction coupling than the control group (Figure 4C). Electrophysiological (EP) studies were also conducted to classify the hiPSC-CM cell types. AP subtypes in hiPSC-CMs were classified as nodal-type (APD90 < 100 ms), atrial-type (100 ms ≤ APD90 < 250 ms), and ventricular-type (APD90 ≥ 250 ms) based on AP duration. Pie charts show the distribution of AP subtypes in vehicle- and 7-AI-treated cells. Representative AP traces illustrate differences in AP morphology among the subtypes. 7-AI treatment increased the proportion of ventricular-type APs while reducing atrial-type populations, suggesting an influence on cardiomyocyte electrophysiological maturation. APD90, action potential duration at 90% repolarization (Figure 4D).

### 7-AI treatment upregulates cardiomyocyte-specific genes and proteins

**Figure 5:**
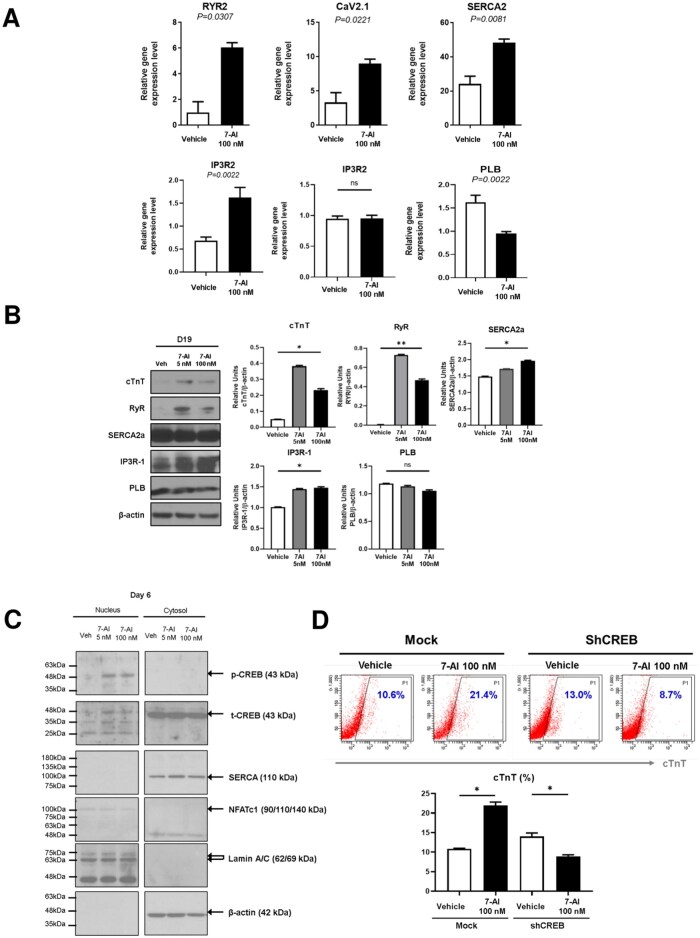
Increased expression of genes and proteins related to cardiomyocyte differentiation and maturation through 7-AI treatment. (A) 7-AI treatment increases the expression of genes involved in calcium handling during cardiomyocyte differentiation. (B) Western blot analysis revealed an increased expression of calcium-handling proteins following 7-AI treatment. (C) Western blot analysis showing the intranuclear migration of CREB, a transcription factor associated with calcium channel regulation, in 7-AI-treated cardiomyocytes. (D) Knockdown of CREB using shCREB inhibited cardiomyocyte differentiation despite 7-AI treatment, indicating the role of CREB in the 7-AI-mediated differentiation process.

Mature CMs efficiently regulate the calcium dynamics. Therefore, we analyzed the expression levels of genes and proteins involved in calcium release, uptake, and storage. Levels of CaV1.2, an L-type calcium channel responsible for calcium influx from the extracellular space into the cytoplasm, and ryanodine receptor 2 and IP3R2, receptors facilitating calcium release from the sarcoplasmic reticulum into the cytoplasm, were significantly higher in the 7-AI-treated groups than in the vehicle group. Levels of SERCA2, which promotes calcium reuptake from the cytoplasm into the sarcoplasmic reticulum, were also elevated. However, no significant difference in the expression levels of sequestrin, a key protein associated with calcium storage, was observed among the groups. Expression levels of phospholamban, which regulates SERCA2 activity and controls the calcium reuptake rate, were lower in the 7-AI-treated groups (Figure 5A). Protein expression analysis revealed significantly increased cTnT and ryanodine receptor levels, slightly increased SERCA2 and IP3R2 levels, and no change in phospholamban levels in the 7-AI-treated groups (Figure 5B). A dose–response analysis using 7-AI at 5, 100, and 500 nM demonstrated that the enhancement of cardiac protein expression plateaued at 100 nM, with no further increase observed at 500 nM. Based on these results, 5 nM was defined as the minimal effective concentration and 100 nM as the optimal concentration, and therefore 100 nM 7-AI was used for subsequent experiments unless otherwise indicated (Figure S1A). Importantly, comparative analysis of cTnT protein expression using other modulators affecting mitochondrial function or cardiomyocyte activity, including cyclosporin A (CsA), endothelin-1 (ET-1), and vitamin C, revealed that although these treatments modestly increased cTnT levels relative to the vehicle control, their effects were markedly lower than those observed in the 7-AI-treated groups. In these conditions, cTnT expression remained comparable to or only slightly higher than vehicle levels, underscoring the superior efficacy of 7-AI in promoting cardiomyocyte-specific protein expression (Figure S1C).

We examined the nuclear translocation of transcription factors responding to calcium signaling to determine the mechanisms underlying the increased expression levels of calcium-related proteins involved in intracellular calcium transport and CM differentiation. To confirm the activation of specific transcription factors during CM differentiation, nuclear and cytoplasmic proteins were separated and analyzed. cTnT transcription factors, including Nkx2.5 (ref.^22^) and GATA4, rely on CREB phosphorylation in response to calcium signaling.^23^ Therefore, we evaluated whether 7-AI treatment induces CREB phosphorylation. Compared to the vehicle, 7-AI increased the nuclear translocation of phosphorylated CREB, even at a low concentration of 5 nM. However, total amount of CREB was unaffected by 7-AI treatment, with most CREB remaining in the cytoplasm. In contrast, NFATc1, another calcium-responsive transcription factor, did not show any increase in nuclear translocation following 7-AI treatment. Relative amounts of nuclear and cytoplasmic proteins were quantified using the nuclear housekeeping protein, lamin A/C, and cytoplasmic housekeeping protein, β-actin, respectively (Figure 5C).

To further assess whether alternative calcium-modulating agents elicit similar nuclear signaling responses, we examined the nuclear and cytoplasmic expression of key cardiac markers, including ryanodine receptor 2 (RYR2) and the cardiac transcription factor NKX2.5, following treatment with cyclosporin A (CsA) or endothelin-1 (ET-1). Compared with vehicle and 7-AI (5 nM)-treated groups, CsA treatment resulted in reduced nuclear and total levels of both RYR2 and NKX2.5, indicating that mPTP inhibition alone is insufficient to recapitulate the nuclear calcium-dependent transcriptional program induced by 7-AI.

In addition, endothelin-1 treatment resulted in detectable changes in myocardial marker expression; however, its effects were qualitatively distinct from those of 7-AI (Figure S1D). Consistent with previous reports, endothelin-1 is not known to induce coordinated upregulation of mature calcium-handling machinery, such as RYR2 and SERCA2, which are hallmarks of functional excitation–contraction coupling in mature cardiomyocytes. Moreover, endothelin-1 predominantly activates hypertrophic signaling pathways rather than nuclear calcium-dependent transcriptional programs associated with cardiomyocyte maturation.^24^ Accordingly, the observed changes are more consistent with partial structural or growth-related responses, in line with the established role of endothelin-1 in cardiac hypertrophic remodeling rather than functional cardiomyocyte maturation.^25^

To further assess the effect of increased nuclear translocation of phosphorylated CREB on CM differentiation of hiPSCs, we used a short hairpin RNA (shRNA) to inhibit CREB expression in the early differentiation stage and subsequently differentiated the cells with or without 7-AI treatment. Flow cytometric analysis was used to count the cTnT-positive CMs and evaluate their differentiation efficiency. In the control group (without CREB knockdown), 7-AI treatment significantly promoted CM differentiation. However, 7-AI treatment did not enhance CM differentiation in the cells treated with the shRNA, which inhibited CREB expression. Notably, vehicle cells showed no significant reduction in differentiation efficiency after CREB knockdown (Figure 5D).

### Correlation between intranuclear calcium transport and cardiomyocyte differentiation and maturation

**Figure 6:**
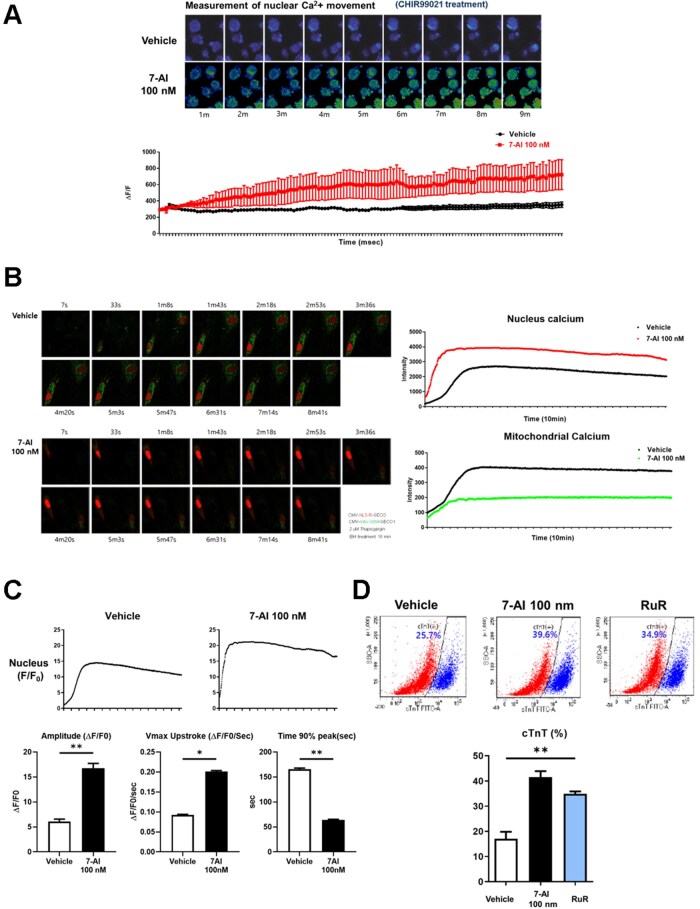
Relationship between increased intranuclear calcium transport and cardiomyocyte differentiation and maturity. (A) Treatment with 7-AI caused an increase in intracellular calcium levels by small molecules during cardiomyocyte differentiation. (B) The movement of the calcium during 7-AI treatment was tracked using a vector that binds to calcium present in the mitochondria and nucleus. In the 7-AI treatment group, mitochondrial calcium migration was inhibited and intranuclear calcium was increased. (C) Treatment with 7-AI enhances intranuclear calcium transport during cardiomyocyte differentiation. (D) Inhibition of calcium influx into the mitochondria by treatment with the MCU blocker Ruthenium Red(RuR) showed almost the same cardiomyocyte differentiation efficiency as 7-AI treatment.

We further investigated the effect of 7-AI treatment on calcium transport to clarify the mechanisms underlying calcium redistribution during the CM differentiation of hiPSCs. In the early differentiation stage, hiPSCs were treated with CHIR99021, a glycogen synthase kinase-3β inhibitor promoting mesoderm progenitor cell differentiation, and calcium movement was monitored using FLUO-4, a calcium-sensitive fluorescent dye. CHIR99021 treatment rapidly increased the cytoplasmic calcium levels. In the 7-AI-treated group, cytoplasmic calcium was transported into the nucleus, resulting in a 3-fold increase in nuclear calcium levels compared to those in the vehicle group (Figure 6A). This increase in nuclear calcium levels was unexpectedly large, prompting us to investigate the underlying mechanisms.

Although 7-AI inhibits mPTP, this effect alone cannot explain the excessive increase in nuclear calcium levels. An earlier version of the 7-AI compound acted as an MCU inhibitor.^26^ Therefore, we hypothesized that MCU inhibition by 7-AI blocks mitochondrial calcium uptake, leading to the accumulation of excess cytoplasmic calcium, which translocates to the nucleus, considerably increasing the nuclear calcium levels. To verify this hypothesis, we used genetically encoded calcium indicators (GECOs), fluorescent indicators changing their fluorescence intensity based on the intracellular calcium levels, to monitor calcium transport over time.^27^^,^^28^ These indicators were targeted to specific cellular organelles. Using vectors obtained from Addgene, we monitored the real-time calcium fluctuations in specific subcellular compartments (Figure 6A).

Differentiated CMs were transfected with the CMV-NLS-R-GECO and CMV-mito-GEM-GECO1 vectors to measure the nuclear and mitochondrial calcium levels, respectively. CMs transfected with these GECO vectors were pretreated with thapsigargin, a SERCA inhibitor blocking calcium reuptake by ER. Next, we added tBH to rapidly increase the intracellular calcium levels and monitored the calcium dynamics in the presence and absence of 7-AI.

Upon tBH treatment, vehicle cells showed calcium influx into both the nucleus and mitochondria, followed by a gradual decrease in calcium levels. In contrast, 7-AI-treated cells exhibited minimal calcium uptake by the mitochondria, and calcium remained concentrated in the nucleus at significantly high levels (Figure 6B). Consistently, quantitative analysis showed that nuclear calcium levels and time to peak intensity were higher in the 7-AI-treated group than in the vehicle group (Figure 6C). These results suggest that 7-AI inhibits mitochondrial calcium uptake, leading to the accumulation of excess cytoplasmic calcium, which translocates to the nucleus, causing nuclear calcium overload.

To validate the hypothesis that MCU inhibition enhances CM differentiation by blocking mitochondrial calcium uptake and promoting nuclear calcium accumulation, we tested whether the effect of 7-AI is replicated by the MCU inhibitor, ruthenium red (RuR). hiPSCs were treated with RuR or 7-AI, followed by the induction of CM differentiation. Flow cytometric analysis of cTnT-positive cells revealed that both the RuR-treated and 7-AI-treated groups showed similar increases in the proportions of cTnT-positive cells, which were significantly higher than that in the vehicle group. These findings suggest that inhibiting mitochondrial calcium uptake increases nuclear calcium accumulation, thereby promoting the differentiation and maturation of hiPSC-CMs (Figure 6D).

## Discussion

This study provides novel insights into the mechanisms by which calcium signaling modulates cardiomyocyte (CM) differentiation and maturation. Generally, 7-AI was known to promote muscle cell maturation by inhibiting mPTP opening and ROS accumulation. Here, we found that 7-AI enhances CM differentiation via an alternative pathway based on intracellular calcium redistribution.

We demonstrated that 7-AI treatment significantly enhanced the differentiation efficiency and functional maturation of both mESC-CMs and hiPSC-CMs. Specifically, 7-AI treatment increased the contractile area, beating rate, and proportion of cardiac troponin T (cTnT)-expressing cells, as revealed by flow cytometry. Additionally, 7-AI-treated groups exhibited morphological changes, including increased cell size and sarcomere length, as well as upregulation of CM-specific markers and ion channel levels.

**Figure 7:**
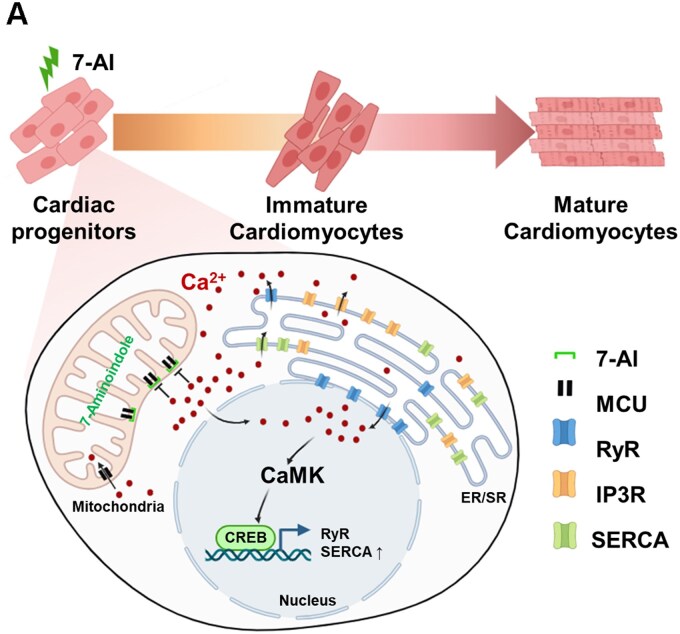
Schematic figure. Effect of the increase of intranuclear calcium by MCU inhibition on the differentiation and maturation of iPSC-derived cardiomyocytes. Schematic illustration showing how 7-AI-mediated inhibition of the mitochondrial Ca^2+^ uniporter (MCU) increases intranuclear Ca^2+^, thereby promoting the differentiation and maturation of iPSC-derived cardiomyocytes. Reduced mitochondrial Ca^2+^ uptake elevates cytosolic/nuclear Ca^2+^ levels and activates CaMK/CREB signaling, which upregulates calcium-handling proteins (eg, RyR, SERCA). This process enhances the transition from cardiac progenitors to immature cardiomyocytes and eventually mature cardiomyocytes. CaMK, Ca^2+^/calmodulin-dependent protein kinase; CREB, cAMP response element-binding protein; ER/SR, endoplasmic/sarcoplasmic reticulum; IP3R, inositol 1,4,5-trisphosphate receptor; MCU, mitochondrial Ca^2+^ uniporter.

Further investigation into the underlying mechanisms revealed that 7-AI primarily inhibited MCU, which facilitates cytosolic calcium uptake by mitochondria. This inhibition prevents excessive mitochondrial calcium accumulation and promotes calcium redistribution to the nucleus. Consequently, nuclear calcium activates the transcription factor CREB, which in turn promotes the expression of key genes involved in CM differentiation and maturation (Figure 7). Similar effects were observed with RuR, another MCU inhibitor, supporting our hypothesis.

Our findings underscore the importance of calcium dynamics in the CM development. While calcium is essential for normal CM function, its dysregulation can lead to various pathological conditions, such as hypertrophy and fibrosis. Interestingly, our study demonstrates that controlled nuclear calcium influx promotes CM differentiation and maturation in progenitor cells without inducing pathological remodeling.

Our findings provide valuable insights for improving the cardiac differentiation protocols. By targeting the MCU and modulating intracellular calcium distribution during the critical cardiac lineage commitment phase, 7-AI enhanced the differentiation efficiency and functional maturation of CMs. These effects should be considered when developing and optimizing functional CM development protocols for applications in regenerative medicine and disease modeling.

Notably, this targeted modulation offers distinct advantages over broad-spectrum chemical inducers, such as 5-azacytidine (5-azaC), which rely on global DNA demethylation and often induce cytotoxicity or off-target lineage effects.^29^ In contrast, 7-AI functions as a “mitochondrial-protective differentiation enhancer”; by preventing mitochondrial calcium overload, it preserves mitochondrial integrity and upregulates structural proteins like Tom20. This creates a robust intracellular environment that naturally favors the metabolic transition toward oxidative phosphorylation (OxPhos) while driving lineage-specific gene expression via the CaMK–CREB axis. Thus, 7-AI enables efficient cardiomyocyte differentiation through precise calcium modulation without the cytotoxicity associated with global epigenetic disruptors.

Despite these developmental benefits, the potential for adverse cellular responses necessitates a cautious approach to 7-AI administration. Although empirical toxicity data specific to 7-AI remains sparse, persistent elevation of nuclear Ca^2+^ has been implicated in chronic cellular strain and homeostatic disruption.^30^^,^^31^ Furthermore, literature on structurally analogous indole derivatives suggests a risk of augmented ROS generation and mitochondrial impairment under conditions of overexposure.^32^^,^^33^ These factors highlight the critical importance of optimizing both dosage and exposure time. To mitigate such risks, we restricted 7-AI treatment to a brief, early window of the differentiation process. This transient intervention successfully catalyzed lineage commitment without compromising cell viability or inducing observable cytotoxicity. Consequently, our findings support the use of 7-AI as a time-delimited molecular switch to enhance differentiation, rather than as a sustained therapeutic agent, ensuring a favorable balance between inductive potency and cellular safety.

Nevertheless, a notable limitation of our study was the lack of in vivo validation experiments. Future studies are essential to confirm the therapeutic potential and safety profile of 7-AI in animal models, thereby bridging the gap between our in vitro findings and the clinical applications in myocardial regeneration.

Future studies should optimize the dosage and timing of 7-AI treatment to maximize its beneficial effects and delineate its impact on other calcium-dependent transcription factors such as NFATc1. Moreover, investigating the effects of 7-AI on the differentiation of other cell types to expand its application as a versatile regulator of calcium dynamics in developmental biology.

## Conclusions

In summary, our study revealed new roles of 7-AI in modulating intracellular calcium distribution to significantly enhance the differentiation efficiency and functional maturation of CMs, highlighting a new avenue for refining cardiac differentiation protocols and advancing myocardial regeneration strategies.

Importantly, this study introduces 7-AI as a promising small molecule modulator of nuclear calcium signaling, offering a novel strategy to fine-tune cardiomyocyte development. Given the simplicity of chemical modulation, these findings have great translational potential for scalable stem cell-based cardiac therapy applications.

## Supplementary Material

szag021_Supplementary_Data

## Data Availability

The data supporting the findings of this study are available from the corresponding author upon reasonable request.

## References

[1] Fujiwara M , Yan P, Otsuji TG, et al Induction and enhancement of cardiac cell differentiation from mouse and human induced pluripotent stem cells with cyclosporin-A. PLoS One. 2011;6:e16734. 21364991 10.1371/journal.pone.0016734 PMC3043062

[2] Birket MJ , Casini S, Kosmidis G, et al PGC-1alpha and reactive oxygen species regulate human embryonic stem cell-derived cardiomyocyte function. Stem Cell Reports. 2013;1:560-574. 24371810 10.1016/j.stemcr.2013.11.008 PMC3871390

[3] Yang X , Rodriguez M, Pabon L, et al Tri-iodo-l-thyronine promotes the maturation of human cardiomyocytes-derived from induced pluripotent stem cells. J Mol Cell Cardiol. 2014;72:296-304. 24735830 10.1016/j.yjmcc.2014.04.005 PMC4041732

[4] Slotvitsky MM , Tsvelaya VA, Podgurskaya AD, et al Formation of an electrical coupling between differentiating cardiomyocytes. Sci Rep. 2020;10:7774. 32385315 10.1038/s41598-020-64581-5 PMC7210299

[5] Lyra-Leite DM , Gutierrez-Gutierrez O, Wang M, et al A review of protocols for human iPSC culture, cardiac differentiation, subtype-specification, maturation, and direct reprogramming. STAR Protoc. 2022;3:101560. 36035804 10.1016/j.xpro.2022.101560 PMC9405110

[6] Feeney AK , Simmons AD, Peplinski CJ, et al Enhancing human pluripotent stem cell differentiation to cardiomyocytes through cardiac progenitor reseeding and cryopreservation. iScience. 2025;28:112452. 40454098 10.1016/j.isci.2025.112452 PMC12124670

[7] Hom JR , Quintanilla RA, Hoffman DL, et al The permeability transition pore controls cardiac mitochondrial maturation and myocyte differentiation. Dev Cell. 2011;21:469-478. 21920313 10.1016/j.devcel.2011.08.008 PMC3175092

[8] Cho SW , Park JS, Heo HJ, et al Dual modulation of the mitochondrial permeability transition pore and redox signaling synergistically promotes cardiomyocyte differentiation from pluripotent stem cells. J Am Heart Assoc. 2014;3:e000693. 24627421 10.1161/JAHA.113.000693 PMC4187507

[9] Park J , Park E, Ahn BH, et al NecroX-7 prevents oxidative stress-induced cardiomyopathy by inhibition of NADPH oxidase activity in rats. Toxicol Appl Pharmacol. 2012;263:1-6. 22659508 10.1016/j.taap.2012.05.014

[10] Kim HJ , Koo SY, Ahn BH, et al NecroX as a novel class of mitochondrial reactive oxygen species and ONOO(-) scavenger. Arch Pharm Res. 2010;33:1813-1823. 21116785 10.1007/s12272-010-1114-4

[11] Li B , Dedman JR, Kaetzel MA. Nuclear Ca^2+^/calmodulin-dependent protein kinase II in the murine heart. Biochim Biophys Acta. 2006;1763:1275-1281. 17069901 10.1016/j.bbamcr.2006.09.029

[12] Sheng M , Greenberg ME. The regulation and function of c-fos and other immediate early genes in the nervous system. Neuron. 1990;4:477-485. 1969743 10.1016/0896-6273(90)90106-p

[13] Ghosh A , Greenberg ME. Calcium signaling in neurons: molecular mechanisms and cellular consequences. Science. 1995;268:239-247. 7716515 10.1126/science.7716515

[14] Puceat M , Jaconi M. Ca^2+^ signalling in cardiogenesis. Cell Calcium. 2005;38:383-389. 16099501 10.1016/j.ceca.2005.06.016

[15] Knollmann BC , Roden DM. A genetic framework for improving arrhythmia therapy. Nature. 2008;451:929-936. 18288182 10.1038/nature06799

[16] Hwang IC , Kim JY, Kim JH, et al Therapeutic potential of a novel necrosis inhibitor, 7-amino-Indole, in myocardial Ischemia-Reperfusion injury. Hypertension. 2018;71:1143-1155. 29661840 10.1161/HYPERTENSIONAHA.117.09405 PMC5959205

[17] Kon N , Murakoshi M, Isobe A, et al DS16570511 is a small-molecule inhibitor of the mitochondrial calcium uniporter. Cell Death Discov. 2017;3:17045. 28725491 10.1038/cddiscovery.2017.45 PMC5511861

[18] Kamer KJ , Mootha VK. The molecular era of the mitochondrial calcium uniporter. Nat Rev Mol Cell Biol. 2015;16:545-553. 26285678 10.1038/nrm4039

[19] Li B , Kaetzel MA, Dedman JR. Signaling pathways regulating murine cardiac CREB phosphorylation. Biochem Biophys Res Commun. 2006;350:179-184. 16996475 10.1016/j.bbrc.2006.09.016

[20] Blayney LM , Lai FA. Ryanodine receptor-mediated arrhythmias and sudden cardiac death. Pharmacol Ther. 2009;123:151-177. 19345240 10.1016/j.pharmthera.2009.03.006 PMC2704947

[21] Baines CP , Kaiser RA, Purcell NH, et al Loss of cyclophilin D reveals a critical role for mitochondrial permeability transition in cell death. Nature. 2005;434:658-662. 15800627 10.1038/nature03434

[22] Keren-Politansky A , Keren A, Bengal E. Neural ectoderm-secreted FGF initiates the expression of Nkx2.5 in cardiac progenitors via a p38 MAPK/CREB pathway. Dev Biol. 2009;335:374-384. 19765572 10.1016/j.ydbio.2009.09.012

[23] Ma H , Groth RD, Cohen SM, et al gammaCaMKII shuttles Ca(2)(+)/CaM to the nucleus to trigger CREB phosphorylation and gene expression. Cell. 2014;159:281-294. 25303525 10.1016/j.cell.2014.09.019 PMC4201038

[24] Sugden PH , Clerk A. Endothelin signalling in the cardiac myocyte and its pathophysiological relevance. Curr Vasc Pharmacol. 2005;3:343-351. 16248777 10.2174/157016105774329390

[25] Lundy SD , Zhu WZ, Regnier M, et al Structural and functional maturation of cardiomyocytes derived from human pluripotent stem cells. Stem Cells Dev. 2013;22:1991-2002. 23461462 10.1089/scd.2012.0490 PMC3699903

[26] Thu VT , Kim HK, Long Le T, et al NecroX-5 prevents hypoxia/reoxygenation injury by inhibiting the mitochondrial calcium uniporter. Cardiovasc Res. 2012;94:342-350. 22425903 10.1093/cvr/cvs122

[27] Wu J , Prole DL, Shen Y, et al Red fluorescent genetically encoded Ca^2+^ indicators for use in mitochondria and endoplasmic reticulum. Biochem J. 2014;464:13-22. 25164254 10.1042/BJ20140931 PMC4214425

[28] Zhao Y , Araki S, Wu J, et al An expanded palette of genetically encoded Ca(2)(+) indicators. Science (1979). 2011;333:1888-1891. 10.1126/science.1208592 PMC3560286 21903779

[29] Gao B , LiY, ZhaoL, et al 5-azacytosine induces cytotoxicity via 5-methylcytosine depletion on chromatin-associated RNA in leukemia. bioRxiv. 2025 Jun 27:2025.06.24.661348.10.1016/j.molcel.2026.08.01842743920

[30] Bagur R , Hajnoczky G. Intracellular Ca(2+) sensing: its role in calcium homeostasis and signaling. Mol Cell. 2017;66:780-788. 28622523 10.1016/j.molcel.2017.05.028 PMC5657234

[31] Gielecinska A , Kciuk M, Kontek R. The impact of calcium overload on cellular processes: exploring calcicoptosis and its therapeutic potential in cancer. Int J Mol Sci. 2024;25:13727. 39769488 10.3390/ijms252413727 PMC11679949

[32] Jiang Y , Fang Y, Ye Y, et al Anti-cancer effects of 3,3′-diindolylmethane on human hepatocellular carcinoma cells is enhanced by calcium ionophore: the role of cytosolic Ca(2+) and p38 MAPK. Front Pharmacol. 2019;10:1167. 31649538 10.3389/fphar.2019.01167 PMC6795059

[33] Filho PCS , da Silva MB, da Silva BNM, et al Seleno-indoles trigger reactive oxygen species and mitochondrial dysfunction in Leishmania amazonensis [in English]. Tetrahedron. 2023;135.

