# Supplementary material for: Effects of increasing intranuclear calcium levels via MCU inhibition on mouse and human PSC-derived cardiomyocyte differentiation and maturation
Source: Stem Cells Transl Med. 2026 Apr 27;15(5):szag021. doi: 10.1093/stcltm/szag021 (PMC13120857; doi:10.1093/stcltm/szag021)
Supplement: szag021_Supplementary_Data [file szag021_supplementary_data.docx]

**Supplementary Information**

**Effects of increasing intranuclear calcium levels via MCU inhibition on mouse and human PSC-derived cardiomyocyte differentiation and maturation**

Hyun Ju Seo^1,8^, Ju-young Kim^3,7,8^, Hyun-Jai Cho^1,2,7,9^*, Joo-Eun Lee^7^, Sang-Beom Bang^1^, Mika Jeon^1^,

Hyang-Ae Lee^4,5^, Yoo-Jeong Shin^6^, Han‑Mo Yang^2^ and Hyo-Soo Kim^1, 2, 7^

Affiliations:

^1^Department of Molecular Medicine and Biopharmaceutical Sciences, Graduate School of Convergence

Science and Technology, and College of Medicine or College of Pharmacy, Seoul National University, Seoul, Korea.

^2^Department of Internal Medicine, Seoul National University Hospital, Seoul, Korea.

^3^ New Drug Development Division, K-BioCELF Inc., Suwon, Korea

^4^ Center for Biomimetic Research, Korea Institute of Toxicology (KIT), 34114, 141 Gajeong-ro, Daejeon, Korea

^5^ School of Korea Institute of Toxicology, University of Science & Technology (UST), 34113, 217 Gajeong-ro, Daejeon, Korea

^6^ Faculty of Medicine and Health, University of Sydney, Sydney, Australia

^7^ Interdisciplinary Program in Stem Cell Biology, College of Medicine, Seoul National University, Seoul, Korea

^8^ These authors contributed equally

^9^Lead contact

*Correspondence: Hyun-Jai Cho, MD, PhD, Department of Internal Medicine, Seoul National University Hospital, Seoul, Korea.

101 Daehak-ro, Jongno-gu, Seoul 03080, Korea. hyunjaicho@snu.ac.kr

**Table S1. mESC-Cardiomyocyte differentiation protocol**

| Day 0 | Aggrewell -EB |  |
| --- | --- | --- |
| Day1-2 | BMP4, Activin A , bFGF 10 ng/mL | EB attache |
| Day 3 | bFGF, EGF 20 ng/mL, CT-1 4 ng/mL, VEGF 5 ng/mL | Media change every two day |
| Day 4 | Same conditions | 7-AI treatment (Day 4~8) |

**Table S2. hiPSC-Cardiomyocyte differentiation protocol**

| Day 0 | 1.5 x 10^6^ cells/dish | MTeSR |
| --- | --- | --- |
| Day 1-2 | CHIR 99021 6μM | RPMI-1640 + B27 |
| Day 4 | Activin A 10 μM, bFGF 20 μM | 7-AI treatment (Day 4~8) |
| Day 6 | IWR1 5 μM | RPMI-1640 + B27 |
| Day 8 | Media change every two day | RPMI-1640 + B27 |

**Supplementary Method 3. mESC culture and differentiation**

mESCs (ES-C57BL/6; ATCC number: SCRC-1002; ATCC, Manassas, USA) were cultured using mouse embryonic fibroblasts (CF-1, ATCC number: SCRC-1040) on an mESC medium with the recombinant mouse leukemia inhibitory factor (ESG1107; Merck Millipore, Darmstadt, Germany). Briefly, 2.2 × 106 mESCs were incubated in an Aggrewell (#27845/27945; STEMCELL Technologies, Vancouver, Canada) in an embryoid body medium with the recombinant bone morphogenetic protein-4 (5020-BP; R&D Systems, Minneapolis, USA) for one day to form embryoid bodies. ESCs were incubated in a suspension culture for two days in embryoid body medium containing 10 ng/mL each of bone morphogenetic protein-4, activin A (recombinant human/mouse/rat activin A 338-AC; R&D Systems), and recombinant human basic fibroblast growth factor (bFGF; 13256029; Thermo Fisher Scientific, MA, USA). On CM differentiation day 3, embryoid bodies were attached to a 6-well plate with CM differentiation medium supplemented 20 ng/mL each of bFGF, recombinant human epidermal growth factor(236-EG; R&D Systems), 4ng/mL recombinant human cardiotrophin-1(612-CD; R&D Systems), and factor 5 ng/mL recombinant mouse vascular endothelial growth (493-MV; R&D Systems), which was changed every two days (Supplementary Table 1). Then, 5 and 100 nM 7-AI was added on CM differentiation days 4–8.

**Supplementary Method 4. Flow cytometry**

mESC-CM and hiPSC-CM differentiation was assessed via flow cytometry using the cTnT marker. Cultured mESC-CMs were detached from the dish using trypsin and washed with phosphate-buffered saline (PBS) via centrifugation at 1800 rpm for 5 min. hiPSC-CMs were dissociated into single cells using accutase and washed with PBS via centrifugation at 1200 rpm for 5 min. These cells were resuspended in 1 mL of fluorescence-activated cell sorting buffer before staining. After permeabilization with the permeabilization buffer (Gibco) at 4 °C for 10 min, the cells were incubated with primary antibodies at room temperature for 1 h, washed with the fluorescence-activated cell sorting buffer, incubated again with the Alexa-488-conjugated secondary antibodies at room temperature for 1 h, and subjected to flow cytometry.

**Supplementary Method 5. Immunofluorescence assay**

To assess the differentiation efficiency of mESC-CMs and hiPSC-CMs after 7-AI treatment, immunostaining was performed using the α-SA Tom20 antibody to visualize the mitochondria. The cells were incubated overnight with primary antibodies at 4 °C, followed by incubation with the secondary Alexa-488- and Alexa-555-conjugated antibodies at room temperature for 1 h. Images were acquired using a confocal microscope (Leica). Cell size was calculated using the mean cell area with the ImageJ software, and cell length was measured in pixels and converted to actual length (μm) for quantitative analysis.

**Supplementary Method 6. Reverse transcription-quantitative polymerase chain reaction (RT-qPCR)**

Next, mRNA expression levels were quantified via real-time RT-PCR using the 7500 real-time PCR system, according to the manufacturer’s protocol. PCR was performed using a 96-well plate with three replicates at a final volume of 20 μL, and expression levels were normalized to glyceraldehyde 3-phosphate dehydrogenase RNA levels. mRNA expression analysis was performed according to the TOYOBO protocol. Quantitative real-time PCR for binding analysis was performed using the SYBR Green protocol.

**Supplementary Method 7 . Western blotting**

Three experimental groups (vehicle, 5 nM 7-AI-treated, and 100 nM 7-AI-treated hiPSC CMs) were lysed using the radioimmunoprecipitation assay buffer containing protease/phosphatase inhibitors and centrifuged at 15,000 rpm for 30 min at 4 °C. The isolated proteins were separated via 10% sodium dodecyl sulfate-polyacrylamide gel electrophoresis and transferred to polyvinylidene difluoride membranes. After blocking with 5% skim milk at room temperature for 30 min, the membranes were incubated with primary antibodies overnight at 4 °C, followed by incubation with the respective secondary antibodies. Immunoblot signals were detected using Amersham 680 and quantified using the ImageJ software.

**Supplementary Method 8. EP study**

Spontaneous action potentials (APs) were recorded from single hiPSC-CMs using current-clamp mode at 37 °C. Cells exhibiting stable spontaneous beating activity were selected for analysis. Once AP wave forms stabilized, the average of five consecutive AP traces was analyzed under each test condition. AP recordings were conducted in an extracellular solution containing (mM) 145 NaCl, 5.4 KCl, 10 HEPES, 1 MgCl2, 5 glucose, and 1.8 CaCl2 (pH 7.4). The internal pipette solution consisted of (mM) 120 K-Asp, 20 KCl, 5 NaCl, 2 CaCl2, 10 HEPES, 5 EGTA, and 5 Mg-ATP (pH 7.25). Cells were monitored to ensure stable electrophysiological properties before data acquisition. AP subtypes in hiPSC-CMs were classified based on action potential duration at 90% repolarization (APD90), the time required for the membrane potential to return to 90% of its resting level following depolarization. Nodal-type APs were defined as APD90 < 100 ms, atrial-type APs as 100 ms ≤ APD90 < 250 ms, and ventricular-type APs as APD90 ≥ 250 ms. Further classification between atrial- and nodal-type APs was based on differences in amplitude (TA) and upstroke velocity (dV/dtmax).

**Supplementary Figure 1.**


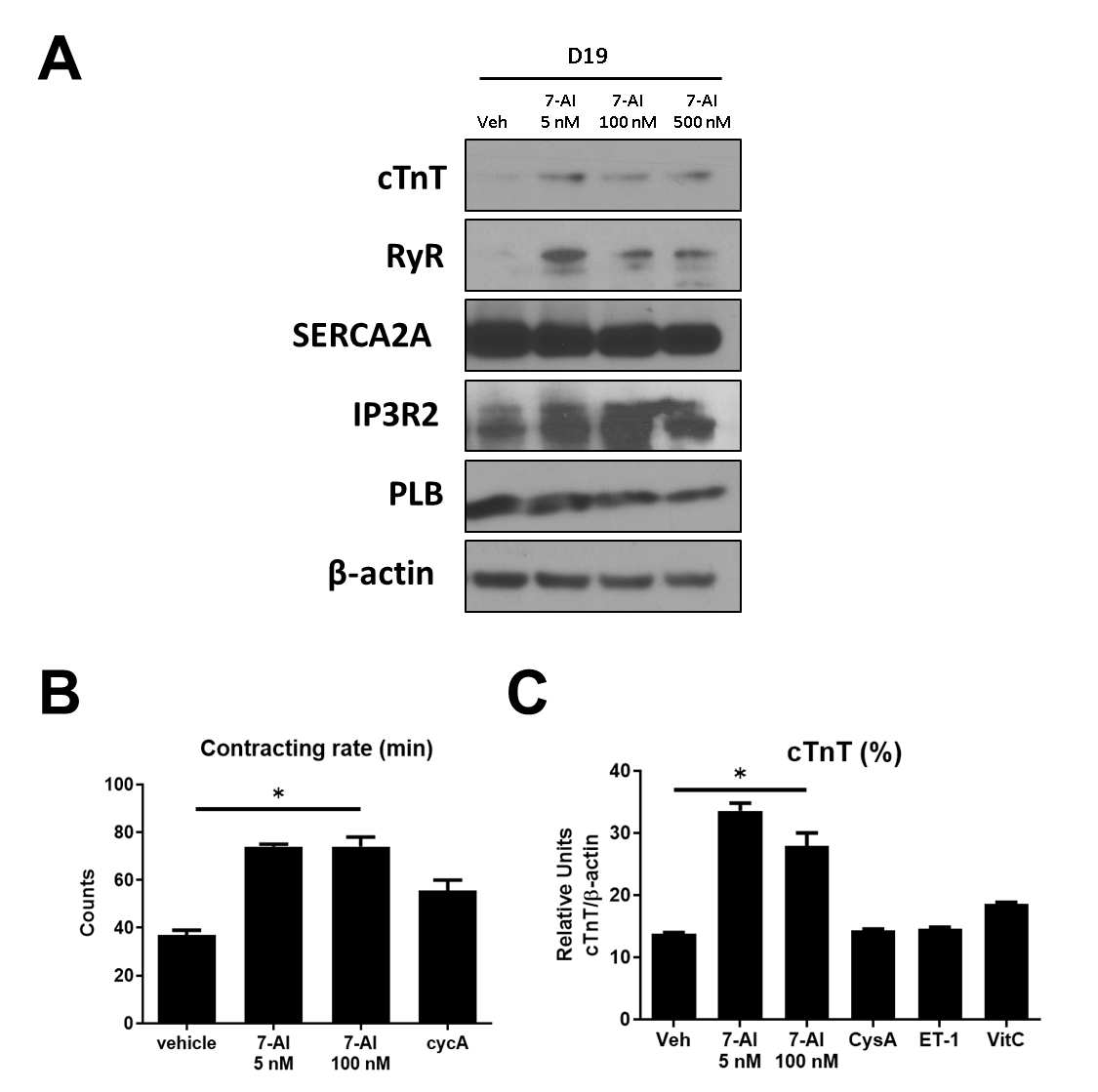


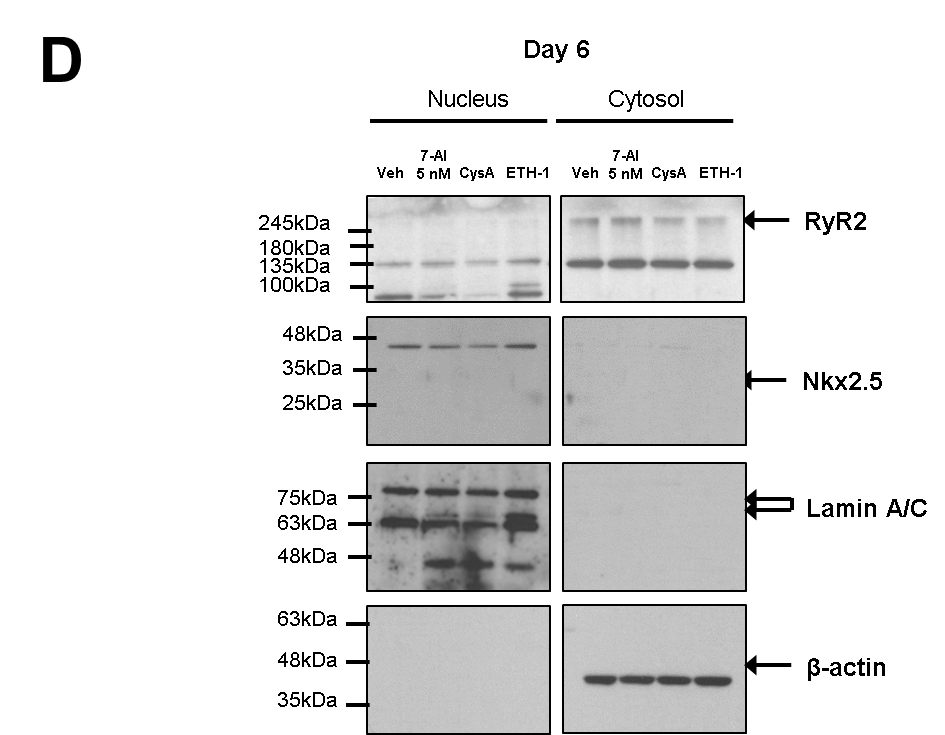


(A) Western blot analysis revealed an increased expression of calcium-handling proteins following 7-AI concentration. (B) Effect of 7-AI on the spontaneous contraction rate of differentiated cardiomyocytes. (C) Quantitative analysis of cardiac troponin T (cTnT) protein expression following treatment with 7-AI and other modulators. (D) Effects of 7-AI on intracellular Ca²⁺-handling proteins and mitochondrial integrity during cardiomyogenic differentiation.
